# Cryptococcid Sweet Syndrome in the Setting of Hydralazine‐Induced ANCA Vasculitis: A Case Report

**DOI:** 10.1111/cup.70044

**Published:** 2025-12-24

**Authors:** Jenna Vroman, Pelin Sagut, Kathryn Lynam, Amanda Ederle, Laura Winterfield

**Affiliations:** ^1^ College of Medicine Medical University of South Carolina Charleston South Carolina USA; ^2^ College of Graduate Studies Medical University of South Carolina Charleston South Carolina USA; ^3^ Department of Dermatology and Dermatologic Surgery Medical University of South Carolina Charleston South Carolina USA

**Keywords:** ANCA, cryptococcoid sweet syndrome, hydralazine, pulmonary and neurological manifestations, vasculitis

## Abstract

Acute febrile neutrophilic dermatosis, also known as Sweet syndrome, is an inflammatory skin condition characterized by the rapid onset of painful, erythematous plaques or nodules with neutrophilic infiltrate on histology. Rarely, acellular bodies surrounded by vacuolated spaces have been noted within the neutrophilic infiltrate, mimicking *Cryptococcus* infection. Despite these histological findings, the cryptococcoid variant of Sweet syndrome is not an infectious process. This delineation is essential for the initiation of proper treatment. Here, we present a patient with cryptococcoid Sweet syndrome with concomitant hydralazine‐induced ANCA vasculitis, which has seldom been reported in the literature.

## 1 Introduction

Acute febrile neutrophilic dermatosis, or Sweet syndrome (SS), is a systemic inflammatory skin condition characterized by fever, leukocytosis, and painful, erythematous plaques and nodules along the face, neck, and upper extremities [1]. Histologically, SS is marked by dense neutrophilic infiltrate and edema in the papillary dermis [1]. A rare histological subtype, cryptococcoid SS (CSS), presents with basophilic, acellular, vacuolated bodies within the neutrophilic infiltrate that bear a resemblance to *Cryptococcus*. However, staining with Grocott/Gomori methenamine silver (GMS) and periodic acid‐Schiff (PAS) fails to reveal organisms.

We present a patient with recurring hydralazine‐induced ANCA vasculitis (HIAV) and CSS with pulmonary and neurological findings. This constellation of findings is rare and highlights the spectrum of neutrophilic dermatoses and leukocytoclastic vasculitis linking HIAV and CSS.

## 2 Case Report

A 67‐year‐old man with a history of end‐stage renal disease (ESRD) on hemodialysis, hypertension, chronic obstructive pulmonary disease, and gastrointestinal (GI) bleeds presented to an outside hospital (OSH) due to shortness of breath and hemoptysis. The patient had no known history of immunosuppression, autoimmune diseases, or dermatologic conditions. Serological tests revealed positive p‐ANCA (1:80), anti‐myeloperoxidase (MPO) antibodies, and anti‐DNase antibodies. The elevated p‐ANCA raised concerns for granulomatosis with polyangiitis (GPA). However, imaging studies demonstrated no pulmonary cavities or nodules. Due to the elevated anti‐DNase antibodies suggestive of HIAV, he was switched from 100 mg of hydralazine three times daily to nifedipine and discharged after a course of IV antibiotics.

Shortly thereafter, he was readmitted to an OSH for potential sepsis and skin lesions. Purpuric papules, plaques, and nodules on forehead, head, arms, and feet were noted on physical exam. Initially treated as disseminated herpes zoster with valacyclovir, lumbar puncture and serological workup were negative for VZV, HSV, monkeypox, cryptococcus, syphilis, and HIV. The patient was switched to doxycycline and prednisone. A dermatology follow‐up two months later revealed no active lesions, and a biopsy showed a reparative reaction.

Months later, he was readmitted to an OSH for suspected pneumonia and an active GI bleed. Chest *x*‐ray revealed bilateral infiltrates consistent with pneumonia. Labs showed a hemoglobin of 30 g/L (Male: 140–170 g/L) [2], which was concerning for an active GI bleed. He was given IV vancomycin and piperacillin‐tazobactam for the pneumonia and transferred to our hospital for management of the GI bleed. Due to limited medical record access from OSH, he was inadvertently restarted on hydralazine. Within 24 h, areas of desquamation from previously healing lesions developed violaceous, edematous bullae on the face and purpuric nodules and plaques on the hands and legs, along with significant facial edema and fever. During this hospitalization, he also developed metabolic encephalopathy, sepsis, and acute hypoxic failure.

**FIGURE 1:**
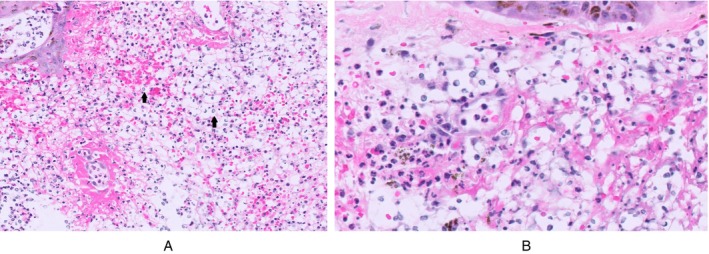
(A and B) Cryptococcoid sweet syndrome. Skin biopsy demonstrating dense infiltration of neutrophils, cellular debris, and numerous basophilic ovoid structures with clear spaces resembling yeast forms (black arrows). (H&E stain, ×20, ×40).

**FIGURE 2:**
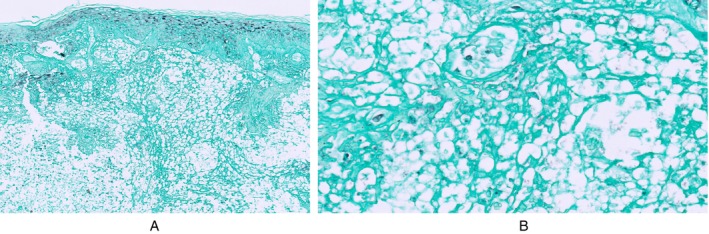
(A, B) Cryptococcoid sweet syndrome. Grocott/Gomori methenamine silver GMS stains of skin biopsy specimen were negative for the ovoid structures at ×10 (2A) and ×40 (2B) magnification.

A skin biopsy specimen revealed a subepidermal blister, epidermal necrosis, neutrophilic infiltrate, and ovoid structures resembling Cryptococcus (Figure 1). GMS stains (Figure 2) and Diastase Chromic Acid Schiff (DCAS) stains were negative. These histological findings, combined with the patient's clinical history, suggested a diagnosis of CSS in the setting of HIAV. Hydralazine was discontinued, and high‐dose corticosteroids were administered, leading to rapid improvement in his symptoms.

## 3 Discussion

Historically, a diagnosis of SS required two major criteria: sudden onset of painful erythematous plaques/nodules and dense neutrophilic infiltrates without evidence of leukocytoclastic vasculitis in histology [1]. However, recent studies propose that vasculitis can occur secondarily to Malone et al. hypothesized that small vessel damage occurs after prolonged exposure to toxic byproducts released by activated neutrophils [3]. Consequently, vasculitic changes on histology should not preclude an SS diagnosis. The specific diagnostic criteria for SS are outlined in Table 1. In addition to both major criteria, the patient was documented to have had at least three of the four minor criteria during his admissions as well (Table 1). Further, the patient had positive p‐ANCA and anti‐MPO antibodies, negative ANA, and rapid deterioration of his vasculitic‐like lesions following hydralazine administration which supports a diagnosis of HIAV. Moreover, the incidence of HIAV is dose dependent [4]. This patient was on 100 mg TID for several years, further strengthening this diagnosis.

**TABLE 1:** Diagnostic criteria for sweet syndrome^a^.

Major criteria
1. Acute onset of tender erythematous plaques or nodules
2. Dense neutrophilic infiltrate on biopsy
Minor criteria
1. Fever, temperature > 38°C
2. Association with an underlying hematologic malignancy, inflammatory disease, or pregnancy, or preceded by an upper respiratory or gastrointestinal tract infection
3. Rapid response to treatment with systemic corticosteroids
4. Abnormal laboratory values at presentation (3 of 4): – Erythrocyte sedimentation rate > 20 mm/h – Positive C‐reactive protein – Leukocyte count > 10 × 10^3^/μL – > 70% neutrophils

^a^ Adapted from Malone et al. [3]. Diagnosis of Sweet syndrome requires both major criteria and two of the four minor criteria to be present.

Given the unusual histopathologic findings in CSS, an expanded differential diagnosis is necessary to enhance diagnostic accuracy. Infectious mimics such as true cryptococcosis, candidiasis, and *Pityrosporum* infection should be considered, particularly in immunocompromised patients or those with comorbid conditions [5]. Other neutrophilic dermatoses, such as pyoderma gangrenosum, bowel‐associated dermatosis‐arthritis syndromes, subcorneal pustulosis, and erythema elevatum diutinum must be considered in patients with ambiguous or atypical presentations [6]. Additionally, due to the clinical overlap, vasculitides like eosinophilic granulomatosis with polyangiits and GPA must be considered [7]. In the setting of underlying infection, as seen in this patient, antibiotic‐induced vasculitis, such as erythema multiforme or erythema nodosum could be on the differential as well [8]. A few reports have described dermatologic manifestations of CSS emerging around the time of antibiotic initiation, particularly vancomycin and piperacillin‐tazobactam, both of which were used in this patient [9]. Although these reports could not definitively determine whether their CSS was triggered by systemic inflammation or represented a drug reaction, Volonté et al. noted the temporal association with antibiotic administration [9]. These mimickers reinforce the importance of accurate history taking, correlating clinicopathologic features, and maintaining a broad differential when evaluating atypical dermal neutrophilic infiltrates.

In the absence of infection, the basophilic acellular bodies seen in CSS are hypothesized to be degrading neutrophils undergoing autolysis and apoptosis [10]. The reason why these cryptococcoid structures form remains elusive. However, there are commonalities among reported cases of CSS. In Table 2, we summarize these findings from 22 documented cases of CSS identified through a PubMed search using the keywords “cryptococcoid,” “Sweet syndrome and vasculitis,” and “Sweet syndrome and Cryptococcus.” Notably, most patients with CSS had one or more of the following comorbidities: underlying malignancy, chronic kidney disease, lung disease, and/or active cocaine use. Among the cases that reported serologic testing, all patients had at least one positive autoantibody. Most frequently, multiple autoantibodies were present.

**TABLE 2:** Clinical and histopathologic findings of reported cases of cryptococcid sweet syndrome.

Reference	Age/sex	Past medical history	Distribution of lesions	Positive serologic autoantibodies	Concomitant vasculitis noted?	Pulmonary and/or neurological involvement?	Medications	Treatment management
Current case	67/M	COPD, ESRD, history of GI bleeds	Face, hands, legs, feet	pANCA, MPO, anti‐DNase	Yes, noted on biopsy	Yes. PNA complicated by hypoxic respiratory failure as well as encephalopathy (both *after* skin lesions).	Hydralazine Acyclovir, doxycycline, vancomycin^a^, piperacillin‐tazobactam^a^	Systemic corticosteroids
Alshaikh et al. [11]	63/M	None	Scalp, face, trunk, upper extremities	Not reported	Not reported	None reported	None	Systemic corticosteroids
Boyd et al. [12]	63/M	ESRD on HD, COPD, CAD, recurrent *Clostridium difficile* colitis	Trunk, extremities	Not reported	Not reported	None reported	“Numerous,” recently started Vancomycin^a^	Metronidazole, topical triamcinolone 0.1% cream
Byekova et al. [13]	82/F	DM, CKD	Mouth, tongue, trunk, extremities	None	Not reported	PNA complicated by respiratory failure	Not reported	N/A (died before improvement noted)
Fletcher et al. [14]	81/M	GI malignancy (neuroendocrine tumor), hypothryoidism, HTN, GERD, an episode of similar cutaenous eruptions 3 years prior	Forehead, cheeks, fingers	Not reported	Not reported	None reported	Not reported	Systemic corticosteroids
Fresco et al. [15]	73/F 50/F	ESRD on HD, DM, CHF ESRD on HD, chronic hepatitis C infection, CAD, cocaine use, one episode of CSS documented 2 months prior	Face, trunk, upper and lower extremities Lower extremities	MPO MPO	Yes, noted on biopsy Yes, noted on biopsy	Respiratory failure and mental status deterioration Mental status deterioration	Not reported Not reported	N/A (died before improvement noted) Systemic corticosteroids
Jordan et al. [16]	81/F 87/F	DM, metastatic breast cancer DM, HTN, CKD	Feet, face, arms Face, trunk, extremities	Not reported Not reported	Not reported Not reported	None reported None reported	Not reported Not reported	N/A (died before improvement noted) Systemic corticosteroids
Ko et al. [10]	76/F 84/F 79/F	AML SLE CLL	Upper and lower extremities Face, lip Face, back, neck	ANA “Multiple autoantibodies” Not reported	LCV noted on biopsy Yes, noted clinically Absent	None reported PNA None reported	Piperacillin‐tazobactam, vancomycin. Piperacillin‐tazobactam^a^ Not reported	N/A (died before improvement noted) Systemic corticosteroids Topical metronidazole
Lim et al. [17]	68/F	DM, ESRD on HD	Face, hands, toes	ANCA	Absent	None reported	Had antibiotics as she was admitted for infective endocarditis but antibiotics were not specified^a^	Systemic corticosteroids
Mazzei et al. [18]	18/F	Pyoderma gangrenosum, Sweet syndrome, ANCA‐positive vasculitis complicated by ESRD on HD	Face, hands	Not reported	History of vasculitis, but not otherwise noted on biopsy.	None reported	Azathioprine, prednisone, losartan, valaciclovir, trimethoprim–sulfamethoxazole	Systemic corticosteroids
Sherban et al. [19]	70/F 68/F 70/F	ESRD, DM, COPD Chronic anemia, HTN, HLD ESRD, DM	Face, neck, chest, upper extremities Face, eyelids, upper extremities, legs Head, upper extremities, tongue	pANCA, PR3 pANCA, MPO, equivocal PR3 MPO, anti‐histone	Absent Yes, noted on biopsy Absent	PNA complicated by respiratory failure None reported Viral PNA	Piperacillin/tazobactam^a^, vancomycin^a^, cefepime^a^, doxycycline^a^, cephalexin^a^, cefazolin^a^ Not reported Not reported	Systemic corticosteroids + dapsone N/A (died before improvement noted) Prednisone, dapsone, mycophenolate mofetil
Skaljic et al. [20]	70/F	HTN, DM, bladder cancer, CKD	Face, arms, legs, mouth	ANA, anti‐histone, anti‐dsDNA, pANCA, MPO	Yes, noted on biopsy	None reported	Hydralazine, amlodipine, clonidine, aspirin, clopidogrel, furosemide, simvastatin, insulin.	Systemic corticosteroids
Stauder et al. [21]	57/M	CKD, HTN, BPH, DM, OSA, HLD, psoriasis, substance use (+remote cocaine use)	Head, trunk, hands, extremities, tongue	ANA, pANCA, anti‐histone, MPO, PR3	Yes, focal vasculitis noted on histology	Hypoxic respiratory failure (prior to skin lesions)	Hydralazine, insulin, amlodipine, aspirin, carvedilol, finasteride, glimepiride, losartan, metoprolol, tamsulosin.	Systemic corticosteroids + dapsone
Volonté et al. [9]	57/M	Anemia due to melena of unknown orgin, pauci‐immune p‐ANCA‐associated crescentic glomerulonephritis leading to ESRD on HD, COPD, HTN, gastritis	Head, tongue, lips, nasal mucosa, trunk, extremities	pANCA, MPO	Yes, focal vasculitis noted on histology.	PNA (prior to skin lesions) Respiratory failure (after skin lesions)	Oral prednisone, escitalopram, bisoprolol, ramipril, doxazosin. piperacillin‐tazobactam^a^, levofloxacin^a^	Systemic corticosteroids
Wilson et al. [22]	48/F 55/F	Anemia, cocaine use Chronic hepatitis C, RA, cocaine use	Neck Face, trunk, extremities, mouth	ANA, pANCA, anti‐SSA pANCA	Absent Absent	None reported None reported	Not reported None	Systemic corticosteroids Systemic corticosteroids
Wilson et al. [23]	75/M	HTN, DM, CAD, ESRD	Face, ears, scalp, hands, elbows	ANA, anti‐histone	Absent	PNA (prior to skin lesions)	Aspirin, atorvastatin, clonidine, hydralazine, insulin, isosorbide mononitrate, lansoprazole, metoprolol. Piperacillin‐tazobactam^a^, vancomycin^a^, ciprofloxacin^a^	Systemic corticosteroids

Abbreviations: AML, acute myeloid leukemia; ANCA, antineutrophilic cytoplasmic antibodies; BPH, benign prostatic hypertension; CAD, coronary artery disease; CHF, congestive heart failure; CKD, chronic kidney disease; CLL, chronic lymphocytic leukemia; COPD, chronic obstructive pulmonary disease; DM, diabetes mellitus; ESRD, end stage renal disease; GERD, gastroesophageal reflux disease; GI, gastrointestinal; HD; hemodialysis; HLD, hyperlipidemia; HTN, hypertension; LCV, leukoclastic vasculitis; MPO, myeloperoxidase; OSA, obstructive sleep apnea; PNA, pneumonia; SLE, systemic lupus erythematosus.

^a^ Patient started on medication during hospitalization but before manifestation of skin lesions.

Importantly, not all patients with CSS exhibited concomitant vasculitis. In those who did, vasculitis was frequently found in patients who had a history of taking hydralazine and/or had recently been given piperacillin‐tazobactam and/or vancomycin in the setting of a pneumonia. These patients commonly showed poor response to antibiotics alone, progressing instead to the development of skin lesions and respiratory failure. Timely initiation of systemic corticosteroids was consistently associated with clinical improvement, both in terms of cutaneous findings and respiratory status. Nevertheless, a few patients failed to respond to treatment and died during their hospitalization.

Although previously reported cases of CSS share several clinical features, to our knowledge, this is the first documented case of CSS with concomitant vasculitis demonstrating both SS‐associated pulmonary and neurologic manifestations. Classic pulmonary manifestations of SS include bilateral infiltrates and pleural effusion imitating pneumonia [24], while neurological manifestations (Neuro‐Sweet disease) include encephalitis and meningitis [25]. We hypothesize that this patient's encephalopathy and respiratory failure may have been exacerbated by CSS.

Systemic corticosteroids and termination of hydralazine effectively improved the patient's condition. Hydralazine was added to his allergy list to prevent future exposure.

Future research of CSS should focus on elucidating its etiology and determining if CSS represents a distinct entity or is merely a histopathologic reaction to systemic stressors. Additionally, outcomes should be compared between individuals with histologic findings of classic SS to those with CSS to determine whether one variant is associated with a worse prognosis.

## Author Contributions

J.V. was the primary author of the manuscript. P.S., K.L., A.E., and L.W. played instrumental roles in drafting and revising the manuscript.

## Ethics Statement

The authors have nothing to report.

## Conflicts of Interest

The authors declare no conflicts of interest.

## Data Availability

Data sharing not applicable to this article as no datasets were generated or analysed during the current study.
